# Prevalence Models to Support Participation: Sensory Patterns as a Feature of All Children’s Humanity

**DOI:** 10.3389/fpsyg.2022.875972

**Published:** 2022-06-23

**Authors:** Evan E. Dean, Lauren Little, Scott Tomchek, Anna Wallisch, Winnie Dunn

**Affiliations:** ^1^Kansas University Center on Developmental Disabilities, University of Kansas, Lawrence, KS, United States; ^2^Department of Occupational Therapy Education, Rush University, Chicago, IL, United States; ^3^Department of Pediatrics, University of Louisville, Louisville, KY, United States; ^4^Juniper Gardens Children’s Project, University of Kansas, Kansas City, KS, United States; ^5^Department of Occupational Therapy Education, University of Missouri, Columbia, MO, United States

**Keywords:** sensory processing, ASD, ADHD, general population, children, sensory profile, participation, environment

## Abstract

**Purpose:**

Research about children tends to consider differences from expected patterns problematic, and associates differences with disabilities [e.g., Autism, attention deficit hyperactivity disorder (ADHD)]. When we focus on disabilities and consider differences automatically problematic, we miss the natural variability in the general population. The International Classification of Functioning, Disability and Health (ICF 11) acknowledges that the experience of disability results from interactions between “environmental” and “personal” factors which determine the person’s capacity to participate. The purpose of this study was to examine sensory patterns across a national sample of children in the general population and samples of children with disabilities to investigate the extent to which differences in sensory processing are representative of natural variability rather than automatically problematic or part of a disability.

**Materials and Methods:**

We employed descriptive statistics and chi-square tests to examine sensory processing patterns in children in the general population and autistic children and children with ADHD. We used standardization and validity data from the Sensory Profile 2 to conduct analyses.

**Results:**

Consistent sensory patterns exist across all groups. Children in all groups had different rates of certain patterns.

**Conclusion:**

Since children in all groups have certain sensory patterns, we cannot associate differences with problematic behaviors. Children participating successfully with all sensory patterns might provide insights for universal design that supports participation of all children.

## Introduction

Research describing children frequently focuses on maladaptive behaviors, associating these behaviors with meeting diagnostic criteria. The International Classification of Function model (ICF 11) (World Health Organization, 2013) invites us to think differently. The ICF makes it clear that “activities” and “participation” are critical features of health. In addition, the ICF acknowledges that disabilities result from an interaction between the “environmental” and “personal” factors which determine the person’s capacity to participate. Consistent with the ICF view, Willis et al. (2017) conducted a scoping review to identify the elements of meaningful participation for children with disabilities and identified “person-based elements” and “environment-focused elements” as substantial factors. Egilson et al. (2017) added to this alignment when they report that autistic children participate less in community activities, and parents report that social and physical environmental features create barriers.

Sensory processing is a factor that bridges personal and environmental factors. Pfeiffer et al. (2018) consider autistic people and describe the lack of fit between personal characteristics such as sensory processing and environmental characteristics (e.g., sensory stimuli) as critical factors in limited or satisfying participation. Other authors have also described lack of fit between individuals’ sensory processing patterns and the sensory environment as instrumental to performance of activities of daily life (Hochhauser and Engel-Yeger, 2010; Reynolds et al., 2011). Chien et al. (2016) found that children with differences in sensory processing, when compared to national norms, had lower participation overall and enjoyed themselves less than children with expected patterns of sensory processing even though both groups participated the same amount. DaLomba et al. (2017) demonstrated a relationship between toddler behavior patterns (using parental perceptions) and patterns of sensory processing. Additionally, Booth et al. (2015) linked high sensitivity to lower satisfaction with life. Therefore, sensory processing, in particular the interaction between the person and the sensory environment, may be an area to inform a more adapted and integrated view about children’s behavior and our approach to supporting their participation.

In this introduction we review the evidence-based concepts of sensory processing and examine how sensory processing has emerged as a critical factor in understanding the person/environment interaction. We propose there is a need to examine our use of sensory processing patterns in light of the ICF’s conception of health and disability so we can support all children to participate successfully in their everyday lives.

### Sensory Processing

Sensory processing refers to how an individual detects and responds to environmental and body stimuli. An individual’s sensory preferences and aversions can both support and inhibit activity participation (Dunn, 2001, 2014; Little et al., 2015). In this way, particular behaviors may reflect adaptive responses based on individuals’ sensory needs even when those behaviors are challenging in a particular context.

Based on Dunn’s Sensory Processing Framework (DSPF; Dunn, 2014), children may exhibit clusters of behaviors that reflect underlying sensory detection thresholds (how quickly one detects) and self-regulation strategies (how one manages input). Sensory patterns include Sensitivity (low threshold, passive self-regulation); Avoidance (low threshold, active self-regulation); Seeking (high threshold, active self-regulation); and Registration (high threshold, passive self-regulation). These patterns were identified from a national sample of children (Dunn, 1999) and have been validated in other studies examining people across the life span (e.g., Daniels and Dunn, 2000; Brown et al., 2001; Dunn et al., 2002; Pohl et al., 2003; Dunn, 2006, 2014).

### Sensory Patterns in Disability Groups

Research about children’s sensory patterns has investigated the ways in which children with various disabilities show higher rates of sensory responses when compared to their peers without conditions (Dunn et al., 2016). For example, studies show that autistic children show higher rates of sensory responses compared to typically developing peers (Baranek et al., 2006; Tomchek and Dunn, 2007). Studies have shown variability in findings with Registration (failing to detect sensory information) (Ben-Sasson et al., 2007) and Seeking (Miller et al., 2007) reported as pronounced sensory patterns in autistic children; however, other research suggests that Avoidance and Sensitivity are highly characteristic of autistic children (Baranek et al., 2007). Sensory processing patterns can also define distinct profiles of autistic children (Lane et al., 2010, 2011, 2014; Ausderau et al., 2014; Tomchek et al., 2018). These studies have consistently identified four subtypes characterized by the overall intensity of sensory patterns within multisensory systems.

Children with ADHD also show sensory sensitivities and avoidance to sensory input (Reynolds and Lane, 2008). Using the Short Sensory Profile (SSP; McIntosh et al., 1999), Mangeot et al. (2001) found that children with ADHD demonstrated higher variability in sensory responses compared to typical peers on all scales of the SSP. Sensory sensitivity and avoidance have been a consistent finding (Mangeot et al., 2001; Lane et al., 2010; Reynolds et al., 2010). Dunn and Bennett (2002), using the Sensory Profile (Dunn, 1999), found that children with ADHD differed from typically developing peers on sensory seeking, emotional reactivity, and inattention-distractibility. In partial support of these findings, Yochman et al. (2004) found that preschool aged children with ADHD differed from those with typical development on Seeking; however, the ADHD group did not significantly differ on Registration. Additionally, Pfeiffer et al. (2015) using the Sensory Processing Measure (Parham and Ecker, 2007), found that children with ADHD demonstrated increased overall sensory processing scores as compared to those without ADHD. These findings are consistent with reports that children with ADHD have reduced processing and scanning linked to cognitive functions (Capri et al., 2020; Mohammadhasani et al., 2020); low threshold sensory patterns (i.e., sensitivity and avoiding) are also associated with high detection paired with low capacity to process sensory input.

### Group vs. Individual Patterns

In group comparisons such as those described above, we may lose sight of the extent to which individual children have differences in sensory processing that affect their everyday routines and activities. Do just some of the children contribute to the statistically significant differences, or are the group findings characteristic of the entire sample? Additionally, because the focus of the above-mentioned studies was to identify patterns that are prevalent in children with specific disabilities such as Autism and ADHD, we lack an understanding of children in the general population. There has been a paucity of research on the extent to which children in the general population may also show particular sensory patterns. If we apply the ICF broadly, we need to understand how sensory processing helps us understand all children’s participation.

Researchers have begun to focus on the variability of sensory processing in children from the general population (Meredith et al., 2015; Little et al., 2018). Emerging evidence suggests that some children in the general population might also display high rates of sensory-related behaviors (e.g., avoidance or sensitivity patterns). For example, researchers have found that avoidance is related to anxiety (Farrow and Coulthard, 2012; Lane et al., 2012; Kotsiris et al., 2020) and associated with sleep difficulties (Shochat et al., 2009) in typically developing children. Similarly, another study found relationships between all four sensory patterns and sleep habits across school-aged typically developing children (Rajaei et al., 2020). Additionally, Registration relates to both easy going approaches and delayed responding to intense situations in typically developing children (DeSantis et al., 2011). Recognizing the variability in sensory processing patterns in the general population, this research encourages researchers and practitioners to think beyond identifying and ameliorating “individual deficits” of sensory preferences and instead focus on environmental and activity features that support participation. In the book *Saving Normal: An Insider’s Look at What Caused the Epidemic of Mental Illness and How to Cure It*, Frances (2013) succinctly sums up the dilemma between identifying individual deficits and supporting participation as:

We must reconcile to there not being any simple standard to decide the question of how many of us are abnormal. The normal curve tells us a great deal about the distribution of everything from quarks to koalas, but it doesn’t dictate to us where normal ends and abnormal begins. Human difference was never meant to be reducible to an exhaustive list of diagnoses. It takes all types to make a successful tribe and a full palette of emotions to make a fully lived life. We shouldn’t medicalize difference and attempt to treat it away (p. 8).

Finally, most previous research has considered each sensory pattern in isolation. It is unclear how many children have 2 or more sensory patterns that are different from the expected “just like others” range (i.e., −1 standard deviation to +1 standard deviation), and whether these children have disabilities (e.g., Autism, ADHD) or are from the general population. If children in the general population who are successfully participating in their lives have two or more sensory patterns in the difference range (i.e., more than 1 standard deviation from the mean in either direction), then we cannot attribute participation challenges of children with disabilities such as Autism and ADHD solely to their sensory pattern differences.

### Sensory Processing and Participation

Dunn et al. (2016) conducted a scoping review about the relationship between sensory processing and participation in everyday activities. They reviewed 261 articles from 122 different journals and included children with (e.g., Autism, ADHD) and without conditions (general population). They reported an increasing pattern of studying the impact of sensory processing on everyday life across a 10-year period. The studies demonstrated a clear relationship between sensory processing and activities of daily living (ADL’s) such as eating (e.g., Coulthard and Blissett, 2009; Marquenie et al., 2011; Nadon et al., 2011) and sleeping (Wengel et al., 2011; Reynolds et al., 2012), instrumental ADL’s (IADL’s) such as school learning (e.g., Brown and Dunn, 2010) and socialization (e.g., Cosbey et al., 2010; Robertson and Simmons, 2013) as well as other aspects of cognition (e.g., Nardini et al., 2008) and temperament (Reynolds and Lane, 2009; DeSantis et al., 2011) that mediate participation outcomes. For example, autistic children who had enhanced perception (i.e., sensory hyperacuity and attention to details) were more likely to have increased activity participation, and children with a high Seeking pattern participated in more adult/child play with family (Little et al., 2015). In ADHD, researchers have shown that children have reduced development of automatic processing, which can impact school learning (Capri et al., 2020; Mohammadhasani et al., 2020). Additionally, in the general population Avoiding and Seeking patterns seem to negatively affect resiliency while Avoiding also negatively affects adaptability. The authors concluded that professionals may need to provide more support for children with Avoiding patterns to overcome obstacles or adjust to changes in routine (Dean et al., 2018). These findings illustrate that all children participate in distinct ways that reflect their sensory patterns, pointing out the need to understand how sensory processing distributes across the population.

The purpose of this study was to examine sensory patterns across a national sample of children in the general population and samples of children with disabilities to investigate the extent to which differences in sensory processing are representative of natural variability in all children rather than automatically problematic or part of a disability.

### Research Questions

This study addressed the following research questions:

(1) What is the distribution of sensory pattern scores among children ages 7 months to 14 years 11 months in the general population?
(2) What is the distribution of sensory pattern scores among autistic children and children with ADHD when compared to the general population?

## Materials and Methods

### Sample

For this study, we used the standardization sample for the Sensory Profile 2 (Dunn, 2014); specifically, we used the data from 1,065 children who were part of the standardization (*n* = 805, 76% of the sample) and children included in validity studies that compared children with conditions to their peers without conditions (*n* = 260, 24% of sample) for the Toddler Sensory Profile 2 (TSP2) and the Child Sensory Profile 2 (CSP2). Table 1 provides a summary of the children’s demographic characteristics.

**TABLE 1:** Summary of the study sample.

Child groups	*N*	%
General population	805	76%
Children with conditions:		
Developmental delay (DD), Intellectual disability (ID), Down syndrome (DS)	25	2%
Autism	70	7%
Autism + ADHD*	22	2%
ADHD*	85	8%
Learning disability	40	4%
Gifted	18	2%
Total	1,065	100%

*n, number of children in each group; %, percentage of total sample.*

*Attention deficit hyperactivity disorder.

### Design

We employed a descriptive design to characterize the patterns of sensory processing in the children included in this study.

### Data Collection Process

The Pearson testing company used their national sites to obtain informed consent for the data for this database. We obtained a de-identified data set for analyses and documented this with our human subject’s office.

### Measures

We used the data from the TSP2 (7–36 months) and the CSP2 (3–14 years, 11 months) measures. These measures are parent reports of the frequency their children respond to sensory events in everyday life. The TSP2 contains 54 items and the CSP2 contains 86 items. Both measures produce scores that align with the four sensory processing patterns described in DSPF (i.e., Avoiding, Registration, Sensitivity, and Seeking). Parents respond to statements about sensory experiences in everyday life by recording how frequently the children engage in that behavior on a 6 point Likert Scale (i.e., 5 = almost always, 4 = frequently, 3 = half the time, 2 = occasionally, 1 = almost never, 0 = does not apply). There is strong validity and reliability for these measures (Dunn, 2014).

The Sensory Profile measures yield category scores based on the bell curve. Expected scores (i.e., “just like others”) include 68% of any group and fall between −1 standard deviation (SD) and +1 SD. Scores reflecting more frequent behaviors are considered “more than others” scores (i.e., scores higher than +1 SD). Scores reflecting less frequent behaviors are considered “less than others” scores (i.e., scores lower than −1 SD). The “more than others” and “less than others” categories each represent approximately 15% of a sample.

### Data Analysis

We used descriptive statistics, chi-square tests, and visual displays of the data to characterize sensory processing patterns of the groups.

## Results

### Research Question 1

**FIGURE 1:**
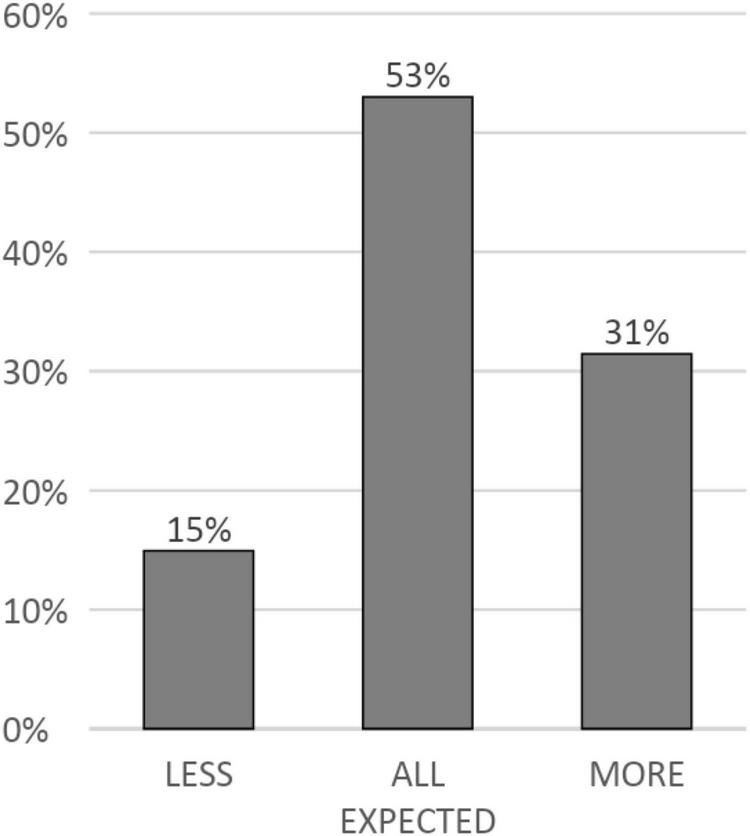
Distribution of “expected,” “more than others,” and “less than others” scores in the general population. Less, all children who had 1 or more sensory pattern scores in the “less than others” categories; More, all children who had 1 or more sensory pattern scores in the “more than others” categories. All Expected, all children who had all 4 sensory pattern scores in the “just like others” category.

The first research question was: “What is the distribution of sensory pattern scores among children ages 7 months to 14 years 11 months in the general population?” We graphed the findings for this question in Figure 1. As you can see, 53% (*n* = 565) of the sample have all 4 of their sensory processing pattern scores in the expected range, 31% (*n* = 327) have one or more sensory processing pattern scores in the “more than others” range, and 16% (*n* = 166) have one or more sensory processing pattern scores in the “less than others” range. Seven children had missing data and could not be included in this analysis.

### Research Question 2

**FIGURE 2:**
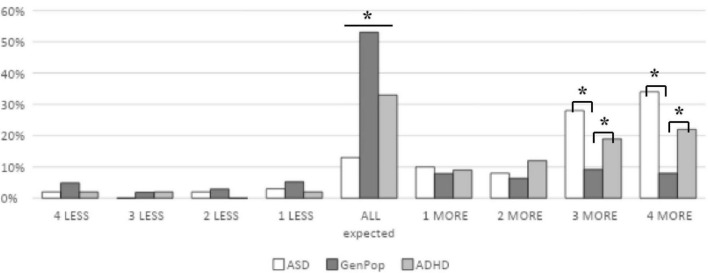
Distribution of sensory pattern scores among three groups of children. ASD, autism; GenPop, general population; ADHD, attention deficit hyperactivity disorder. *Denotes significant differences at *p* < 0.05.

The second research question was: “What is the distribution of sensory pattern scores among autistic children and children with ADHD when compared to the general population?” (Figure 2). As you can see, 13% (*n* = 8) of autistic children, 32% (*n* = 28) of children with ADHD and 53% (*n* = 565) of children in the general population group have all 4 of their sensory processing pattern scores in the expected range. Additionally, 34% (*n* = 21) of autistic children, 22% (*n* = 18) of children with ADHD and 8% (*n* = 85) of children in the general population group have all 4 of their sensory processing pattern scores in the “more than others” range. Fewer children had all 4 scores in the “less than others” range; 2% (*n* = 1) autistic children, 2% (*n* = 2) children with ADHD and 5% (*n* = 52) children in the general population.

To examine if the distribution of scores between autistic children, children with ADHD, and those in the general population was significantly different, we used chi-square analyses. Results showed significant differences in the number of sensory patterns that fell within the “expected” and “more than others” range. We did not use a chi-square to examine the distribution of those that showed sensory processing patterns in the “less than others” range because there were 4 children or less in each cell in the autistic and ADHD groups. First, the three groups (autistic, ADHD, general population) were significantly different in the number of children that had all sensory patterns within the “expected” range, X^2^ (2, *N* = 1065) = 80.99, *p* < 0.001. Using follow up tests with Bonferroni corrections, results showed that children in each group significantly differed from one other (see Figure 2). Second, the number of sensory response patterns that fell within the “more than others” range significantly differed by group, X^2^ (8, *N* = 1065) = 241.26, *p* < 0.001. Using follow up tests with Bonferroni corrections, results showed that children in the general population were significantly different from the autistic and ADHD groups in showing 3 and 4 sensory processing patterns in the “more than others” range. However, the three groups were not different in showing 1 or 2 sensory processing patterns within the “more than others” range. This means that children in the general population and those with autism and ADHD show similar rates of differences in 1 or 2 sensory processing patterns, while autistic children and children with ADHD show higher rates of differences that fall within 3 or 4 sensory processing patterns.

To examine the “more than others” groups in more detail, we investigated which patterns were most prominent within the groups (see Table 2). For the children in the general population group, 27 (3% of total general population group) had only “Seeking” in the “more than others” range, 24 (3% of total general population group) had all 4 sensory processing pattern scores in the “more than others” range.

**TABLE 2:** Number of children who had some pattern of “more than others” scores.

More than others scores	No. of patterns in “more than others” range (more than + 1 SD)	Groups		
		Gen. pop.	Autism	ADHD
All 4 More than others	4	24**	21*	18*
Avoid Seek Sens More	3	7	5	3
Reg Avoid Seek More	3	10	0	2
Reg Avoid Sens More	3	14	11**	8**
Reg Seek Sens More	3	9	4	3
Avoid Seek More	2	6	0	1
Avoid Sens More	2	9	4	1
Reg Avoid More	2	8	0	3
Reg Seek More	2	11	0	1
Reg Sens More	2	7	1	3
Seek Sens More	2	6	0	1
Avoid More	1	16	2	4
Reg More	1	8	2	1
Seek More	1	27*	0	1
Sens More	1	10	2	2
Total with “more than others” scores		172	53	52
% of the group totals		21%	76%	61%

*Highest number of children in group. **Second highest number of children in group. avoid, avoiding score; seek, seeking score; sens, sensitivity score; reg, registration score. Other groups were excluded from this analysis (DD, ID, DS, learning disability, and gifted).

For the children in the autistic group, 22 (31% of total autistic group) had all 4 sensory processing pattern scores in the “more than others” range, and 11 (16% of total autistic group) had 3 pattern scores (i.e., Registration, Sensitivity, Avoiding) in the “more than others” range.

For the children in the ADHD group, 18 (21% of total ADHD group) had all 4 sensory processing pattern scores in the “more than others” range, and 8 (9% of total ADHD group) had 3 pattern scores (i.e., Registration, Sensitivity, Avoiding) in the “more than others” range. Other groups (Learning Disabilities, Gifted, Developmental Delay) had small numbers and so were excluded from the “more than others” analysis.

## Discussion

This study is the first to examine the prevalence of sensory patterns in the general population. The findings illustrate that children in the general population, as well as children with disabilities, exhibit differences in expected sensory patterns. Therefore, we cannot associate those patterns solely to disability groups. Additionally, many children with disabilities scored within the “expected” ranges based on the standardization sample on all four sensory processing patterns. These results demonstrate the importance of considering individual responses to sensory stimuli instead of generalizing based on particular conditions. We will discuss key points here.

### Implications for Supporting Participation

As detailed below, evidence from this study indicates that children with and without identified conditions have sensory processing scores both within the expected range and ranges outside of the expected range. This suggests that participation may be broadly supported for all children by contextual interventions (e.g., adapting places and tasks to meet the sensory preferences) and universal design that provides a way to participate for everyone (Dean et al., 2019). Further, this research adds weight to the argument that we need to normalize rather than pathologize sensory preferences outside of the expended range and focus instead on building supports for participation. There is a need for research focused on understanding the strategies that children use to successfully participate in environments that do not match their sensory preferences, which can inform practitioner strategies for supporting children who have not yet learned to participate successfully in those same environments.

### Some Children in All Groups Have All “Expected” Scores

Some children in all the groups (i.e., general population, autistic, ADHD) had all 4 patterns of sensory processing scores in the expected range (i.e., between −1 and +1 standard deviation from the mean). As expected, children in the general population were the most likely to have this pattern, although only 61% of them have this profile. Since the standardization cut scores are based on standard deviations, we would expect to see about 68% in the expected score range for each of the 4 sensory processing pattern scores.

Researchers have reported that only about half of children with ADHD have sensory processing as a correlate of their learning challenges (Dove and Dunn, 2008); we see in our data that 32% (*n* = 27) of children with ADHD have all expected scores on the sensory profile. This finding suggests that we must differentiate the underlying features for children with ADHD to design the most effective interventions to support their learning and participation.

The literature contains many reports about the sensory processing differences of autistic children (Baranek et al., 2006; Tomchek and Dunn, 2007) and ADHD (Parham and Ecker, 2007; Reynolds and Lane, 2008). This study points out that even though group studies report significant differences, there are some children with these conditions whose sensory patterns are in the expected range. Perhaps for children with conditions such as Autism and ADHD who have sensory processing scores in the expected range, other factors are interfering with participation, such as cognitive or psychosocial factors not related to sensory processing. Alternatively, these children could face situations that provide a more intense sensory experience than they are equipped to handle even with expected patterns. Consider a situation that would overwhelm an otherwise calm person, such as a fire drill or a family reunion. It is important to remember that a person with any sensory processing pattern can reach a limit within a particular context.

We also need to examine other features (e.g., demographic variables such as age, cognition) for the children with disabilities that we would expect to have sensory processing differences, but who have all expected scores on the SP2. In our sample, we verified there was no relationship between age and having all expected scores in the autistic or ADHD groups. This finding contrasts with other data which suggests older children are more adaptable (Kern et al., 2006; Ben-Sasson et al., 2009).

### Some Children in All Groups Have at Least One “More Than Others” Scores

Even though children with disabilities are more likely to have differences in their sensory patterns (76% of autistic children and 61% of children with ADHD, see Table 2), 21% of children in the general population group have at least 1 “more than others” score as well. These data make it hard to suggest that “more than others” scores are indicators of a problem. In fact, 3% of the general population children have all 4 sensory processing patterns in the “more than others” range. Recent research studying children in the general population have found children with Avoiding and Seeking patterns also have protective factors, such as resilience and adaptability (Dean et al., 2018). It would be useful to observe and interview children who have “more than others” scores and who are doing well in school and at home to identify the strategies they use to manage their detection and responsiveness to sensory events. Perhaps their methods for adaptation would also be helpful to children who have not figured out how to manage their daily lives as successfully.

### Twice as Many Children Exhibit “More Than Others” Behaviors Than “Less Than Others” Behaviors

Another interesting observation is that in the overall sample, twice as many children have “more than others” scores (*n* = 327) than have “less than others” scores (*n* = 166). There seem to be 2 hypotheses for this finding. First, it might be that the items on the Sensory Profile 2 are worded in such a way that they foster a bias toward the “more than others” responses. Studies about sensory processing have reflected a larger theme of behaviors that are more noticeable; since the SP2 asks about frequency of behaviors, it might be that parents and professionals pay more attention to these noticeable behaviors. Secondly, it might also be true that the groups of children with disabilities that researchers study the most (e.g., Autism, ADHD) are children who exhibit more frequent sensory responding behaviors, and so the scores reflect our attention to those groups rather than all possibilities.

### Some Patterns of Sensory Processing Are More Likely to Occur

It is not surprising based on the literature that children with disabilities are most likely to have a predominance of “more than others” scores (76% for autistic children, 61% for children with ADHD). Consistent with previous literature, 47% of autistic children and 30% of children with ADHD have 3 (with Registration, Avoiding and Sensitivity as primary pattern) or 4 sensory processing pattern scores in the “more than others” range (Kern et al., 2006; Reynolds and Lane, 2008; Ben-Sasson et al., 2009). Many contexts and activities contain sensory features that are likely to be overwhelming for most autistic children or children with ADHD, leading to behaviors related to attention, persistence, withdrawal and/or distractibility. There may be a relationship between sensory patterns and display of automatic responses such as eye movement patterns and attentional processing in ADHD (Capri et al., 2020; Mohammadhasani et al., 2020); specifically, if one’s tendency is to detect more sensory input because of low thresholds, this tendency might result in what appears to be random eye movements and unexpected attentional shifts to “notice” all the input without filtering. Previous research has shown that while autistic children and children with ADHD show heighted responses to sensory stimuli, those with ADHD demonstrate significantly increased rates of visual processing as compared to autistic children and typical development (Little et al., 2017).

If we consider the sensory processing patterns of Registration, Avoiding, and Sensitivity all being in the “more than others” range in more depth, questions can certainly arise about how Registration (a high threshold pattern) fits in with Sensitivity and Avoiding (low threshold patterns). One might expect to see a co-occurrence of Sensitivity and Avoiding as they both reflect a high noticing/responding behavioral profile, and the literature has reported many hyper-responsive behavior patterns for autistic children and ADHD (Baranek et al., 2007; Reynolds and Lane, 2008). But how does Registration fit in?

We gained some insights from the TSP2 standardization data and from the adult literature on sensory processing. On the Registration score on the TSP2 there are 3 items that one might consider inappropriate based on our knowledge about Registration during the first edition of the Sensory Profile (Dunn, 1999). However, when examining the data from these items, they clearly loaded with Registration most strongly and did not load with other sensory patterns (Dunn, 2014). When looking at the adult literature, there is a repeating pattern of Sensitivity and Avoiding having moderate relationships with features such as anxiety, post-traumatic stress and pain catastrophizing (Engel-Yeger and Dunn, 2011a,b,c; Engel-Yeger et al., 2013). In these studies, Registration also has a low but significant correlation with anxiety, post-traumatic stress and pain catastrophizing, with Seeking being unrelated. What could this mean? One hypothesis is that people who tend to miss cues (the behavior profile for people with a “more than others” score on Registration) will eventually notice a potentially challenging stimulus, but by the time they notice, the situation requires immediate action due to the delay in noticing/responding. People with low thresholds notice quickly and take action quickly. When an individual experiences a delay in noticing sensory stimuli, their actions could look similar to noticing early and acting in a big way because of low thresholds. In a latent profile analysis, researchers called this pattern “Mellow…until” to reflect the delay in noticing (mellow part) along with the eventual big response (…until part) (Little et al., 2018).

Children in the general population group were most likely to have only the Seeking score “more than others” (3% of total group) with the other 3 scores in the expected range. Seeking behaviors provide a means for children to gather information and subsequently learn how their bodies (i.e., person factors) work within their contexts (i.e., environmental factors). It would be interesting to study this group of children to see if they are more adaptable, have more insights, are more creative or design solutions differently from their peers with all expected scores.

### Limitations and Future Directions

We did not incorporate additional demographic data into our analyses that might provide a more detailed profile about children with particular sensory patterns. We also focused on the “more than others” categories since these were more prominent in the data; another analysis might investigate the characteristics of children in the “less than others” groupings in more detail. Finally, sensory processing in this study was measured using a standardized parent report measure. While this measure is widely used in research and practice, research using other experimental methods could add new insights into the sensory experiences of the groups of children who participated in this study.

The findings from this study suggest that understanding how children in the general population with differences in sensory processing determine the strategies they use to manage themselves in everyday life is a critical area for research. Insights from these children could provide a way to understand the person/environment interaction for creating universally designed contexts to support all children’s participation. Additionally, these strategies might highlight the importance of considering the impact of the context on expression of sensory patterns.

## Conclusion

Sensory processing provides a bridge between person and environmental factors. This study illustrated that individualized sensory patterns occur in all children. Our findings call into question the practice of saying that sensory processing differences (i.e., “more than others,” “less than others”) alone indicate a problem, deficit or disability. Children with conditions such as autism and ADHD do seem to exhibit certain patterns more frequently than their general population peers. We propose that children in the general population with differences in sensory patterns can be a source of insights about effective methods for managing everyday life successfully. Our findings suggest that adaptation (of activities and environments) based on a child’s sensory patterns may be a powerful vehicle to successful participation, creating a more inclusive context for children with disabilities. When children, their families and professionals understand sensory patterns as a critical feature of person/environment interaction, these insights expand opportunities for learning, development, participation and health.

## Data Availability Statement

The data analyzed in this study is subject to the following licenses/restrictions: The dataset is owned by the publisher of the sensory profile: Pearson Publishing. Requests to access these datasets should be directed to https://www.pearsonassessments.com/store/usassessments/en/Store/Professional-Assessments/Motor-Sensory/Sensory-Profile-2/p/100000822.html.

## Ethics Statement

The studies involving human participants were reviewed and approved by the Institutional Review Board, University of Kansas Medical Center. Written informed consent to participate in this study was provided by the participants’ legal guardian/next of kin.

## Author Contributions

All authors listed have made a substantial, direct, and intellectual contribution to the work, and approved it for publication.

## Conflict of Interest

WD is the author of the Sensory Profile 2; she does not own the copyright for the material but does receive a royalty for the sale of this assessment. The remaining authors declare that the research was conducted in the absence of any commercial or financial relationships that could be construed as a potential conflict of interest. The authors declare that this study received funding from Pearson Publishing to ED. The funder had the following involvement in the study: they provided research assistant support for the original standardization data collection and used their network to identify participants.

## Publisher’s Note

All claims expressed in this article are solely those of the authors and do not necessarily represent those of their affiliated organizations, or those of the publisher, the editors and the reviewers. Any product that may be evaluated in this article, or claim that may be made by its manufacturer, is not guaranteed or endorsed by the publisher.
